# Short-chain fatty acids regulate erastin-induced cardiomyocyte ferroptosis and ferroptosis-related genes

**DOI:** 10.3389/fphar.2024.1409321

**Published:** 2024-07-12

**Authors:** Xiaojun He, Qiang Long, Yiming Zhong, Yecen Zhang, Bei Qian, Shixing Huang, Lan Chang, Zhaoxi Qi, Lihui Li, Xinming Wang, Xiaomei Yang, Wei Dong Gao, Xiaofeng Ye, Qiang Zhao

**Affiliations:** ^1^ Department of Cardiovascular Surgery, Ruijin Hospital, Shanghai Jiaotong University School of Medicine, Shanghai, China; ^2^ Department of Anesthesiology, Qilu Hospital, Cheeloo College of Medicine, Shandong University, Jinan, China; ^3^ School of Medicine, Cheeloo College of Medicine, Shandong University, Jinan, China; ^4^ Department of Cardiology, Johns Hopkins School of Medicine, Baltimore, MD, United States; ^5^ Department of Anesthesiology and Critical Care Medicine, Johns Hopkins University School of Medicine, Baltimore, MD, United States

**Keywords:** short-chain fatty acids, cardiomyocytes, ferroptosis, ischemia/reperfusion injury, ATF3

## Abstract

**Background:**

Ferroptosis has been proven to contribute to the progression of myocardial ischemia/reperfusion (I/R) injury and can be inhibited or promoted by ATF3. Short-chain fatty acids (SCFAs) have shown benefits in various cardiovascular diseases with anti-inflammatory and antioxidant effects. However, the impact of SCFAs on ferroptosis in ischemic-stimulated cardiomyocytes remains unknown. This study aimed to investigate the effect of SCFAs on cardiomyocyte ferroptosis, the expression of ATF3, and its potential upstream regulators.

**Methods and results:**

The expression of ATF3, ferroptosis pathway geneset (*FPG*), and geneset of potential regulators for ATF3 (*GPRA*, predicted by the PROMO database) was explored in the public human myocardial infarction single-cell RNA-seq (sma) dataset. Cardiomyocyte data was extracted from the dataset and re-clustered to explore the FPG, ATF3, and GPRA expression patterns in cardiomyocyte subclusters. A dose-dependent toxic experiment was run to detect the suitable dose for SCFA treatment. The erastin-induced ferroptosis model and hypoxia-reoxygenation (H/R) model (10 h of hypoxia followed by 6 h of reoxygenation) were adopted to assess the effect of SCFAs via the CCK8 assay. Gene expression was examined via RT-PCR and western blot. Ferroptosis markers, including lipid peroxides and Fe^2+^, were detected using the liperfluo and ferroOrange probes, respectively. In the sma dataset, upregulated ferroptosis pathway genes were mainly found in the infarction-stimulated cardiac cells (border zone and fibrotic zone), particularly the cardiomyocytes and adipocytes. The ATF3 and some of its potential transcription factors (*VDR, EGR3*, *PAX5*, and *SP1*) can be regulated by SCFA. SCFA can attenuate erastin-induced lipid peroxidation in cardiomyocytes. SCFA treatment can also reverse erastin-induced Fe^2+^ increase but may strengthen the Fe^2+^ in the H/R model. We also precisely defined a ferroptosis subcluster of cardiomyocytes (CM09) that highly expressed *FPG*, ATF3, and *GPRA*.

**Conclusion:**

The ATF3 and the ferroptosis pathway are elevated in cardiomyocytes of injury-related cardiac regions (border zone, ischemic zone, and fibrotic zone). SCFA can attenuate cardiomyocyte ferroptosis and regulate the expression of ATF3. Our study offers novel insights into the potential targets of SCFAs in the cardiovascular system.

## Introduction

Cardiovascular disease remains the leading cause disease of death worldwide annually. Despite progress in acute treatment, the effectiveness of therapies aimed at reducing the progress of heart failure has been limited due to an incomplete understanding of remodeling processes (Niccoli et al., 2019). Over the last 10 years, ferroptosis, an iron- and lipid-dependent form of regulated cell death, has been recognized as an important process involved in numerous cardiovascular diseases (Fang et al., 2023). The inhibition of ferroptosis and chelation of iron during acute and chronic myocardial ischemia/reperfusion (I/R) injury can result in cardio-protection, highlighting ferroptosis as a potential therapeutic target in myocardial I/R injury (Conrad and Proneth, 2019; Fang et al., 2019; Han et al., 2023). Therefore, thoroughly understanding the mechanisms involved in regulating ferroptosis in cardiomyocytes might improve disease management.

The gene *ATF3* (Activating Transcription Factor 3) has been upregulated in cardiomyocyte subtypes activated by myocardial infarction (MI) stimulation (Kuppe et al., 2022). Studies have indicated that ATF3 can function as a cardioprotective molecule, (Ke et al., 2023), elevated at the early stage of cardiac reperfusion, and inhibit cardiomyocyte ferroptosis triggered by erastin and RSL3 (Liu H. et al., 2022). However, ATF3 also has the ability to promote ferroptosis, (Wang et al., 2020; Fu et al., 2021), indicating its complex role as a ferroptosis regulator.

Short-chain fatty acids (SCFAs) are the main product of fiber fermentation by the gut microbiota and have been shown to protect against myocardial ischemia and I/R injury, (Yu et al., 2021; Zhou et al., 2021), but the underlying mechanisms remain to be elucidated. SCFAs can be absorbed into the bloodstream and play important roles in various physiological processes, such as metabolism, gut barrier function, immune regulation, and inflammation (Yang et al., 2020). The glutathione (GSH) synthesis plays an important role in regulating ferroptosis (Kang et al., 2023). Studies have shown that sodium acetate can reverse the increased level of plasma GSH induced by nicotine in rats, (Dangana et al., 2020) and sodium butyrate was reported to aggravate lipid peroxidation in a high-fat diet (HFD)-fed rats, (Oyabambi and Olaniyi, 2023) both of which indicate the impact of SCFAs on ferroptosis. Butyrate has been reported to ameliorate ferroptosis in ulcerative colitis by modulating the Nrf2/GPX4 signal pathway (Chen et al., 2024). However, whether SCFAs benefit against myocardial ischemia and I/R injury was mediated by the regulation of ferroptosis remains unknown.

Given the emerging evidence of a link between ferroptosis and cardiomyocyte injury, there is a need to investigate the impact of SCFAs on ATF3 expression, as well as its potential regulators, and their role in modulating ferroptosis in cardiomyocytes. This study aims to address this knowledge gap and provide further insights into the therapeutic potential of SCFAs in attenuating cardiomyocyte injury and regulating the occurrence of ferroptosis. We provide expression patterns of ferroptosis pathway genes at single-cell resolution based on public human myocardial infarction. Besides, the effects of SCFA on ferroptosis and ATF3 mRNA levels in cardiomyocytes were explored.

## Methods

### Analysis of single-nucleus RNA sequencing (snRNA-seq) data

The processed spatial multi-omic atlas data (sma) “All-snRNA-Spatial multi-omic map of human Myocardial infarction” was downloaded from the cellxgene database (https://cellxgene.cziscience.com/collections/8191c283-0816-424b-9b61-c3e1d6258a77), and was analyzed by the Seurat (v4.3.0) R package. Cardiomyocyte data was extracted and normalized, followed by principal component analysis (PCA) reduction, batch effect correction with the harmony package, and clustering using Seurat’s FindNeighbors and FindClusters function. The Unified Manifold Approximation and Projection (UMAP) was created via Seurat’s RunUMAP function. The weighted correlation network analysis (WGCNA) was taken by the hdwgcna R package (Morabito et al., 2021; Morabito et al., 2023). Pseudotime trajectory analysis was taken by the Monocle2 R package (Trapnell et al., 2014; Qiu et al., 2017a; Qiu et al., 2017b). The cell-cell communication was analyzed by the cellchat R package (Jin et al., 2021/02).

The ferroptosis-related genes in KEGG hsa04216 (https://www.kegg.jp/dbget-bin/www_bget?hsa04216) and wikipathways WP4313 (https://www.wikipathways.org/pathways/WP4313.html) were combined as a single geneset. We then calculated the z score (Amrute et al., 2023) of this ferroptosis pathway geneset across cardiac regions and cell types in the sma datasets. The heterogeneity distribution of the ferroptosis pathway geneset was observed.

Potential transcription factors of *ATF3* were predicted by the PROMO database (http://alggen.lsi.upc.es/cgi-bin/promo_v3/promo/promoinit.cgi?dirDB=TF_8.3) with species restricted to humans. The promoter region of *ATF3* was defined as the 2000 upstream bases and 100 downstream bases of the ATF3 gene sequence (hg38_knownGene_ENST00000341491.9, range = chr1:212606761-212620875) and acquired from the UCSC database (https://genome.ucsc.edu/) (Table 1).

**TABLE 1:** ATF3 promoter sequence used for transcription factor prediction.

ATF3 promoter sequence
>hg38_knownGene_ENST00000341491.9 range = chr1:212606761-212620875 5’pad = 0 3’pad = 0 strand = + repeatMasking = none GAGATAACAAATAACTTCATTCAAATGCAAACACTCCTCCACCTAATCCCGCCCGGTGTCCGCCGGGCTGCTCCGACACGCCCGGGGTTTACCTGCGCGCACTCCAGCGGGAGGGCGGGTTGTGGAGGTGTGCTGAGCGGCGCGCGGGGGTGAGGGCGTGGAAGCGGAGGGTGGGGCCCGGAGAGCCGTTACCAGGGCGAAAAGTAAAGCGAAAACACCCGCCCTGCACTTCCCGCGCGACGCCGCTGGAAATCGGTTCAGGTCCAGAGCAGGATCTCGGAGGATCCCGCGTGGAACTCCAGGGCTCCCGGGTCCGCCGGGGCGCAAAGACTTCCGAGGCCGCCCTCCGCGTGTTCCCAGGCCCGTGGAGAGGTGGGTGGTCTGAGTGAGGTCGGGCTTGGCGGCGAGGAACCCCGGTGGGGGGAACTGGGGACTTCAAGTGAGACCCAGGCTCCAGACACCTCTAGTTTCTACCCCAAATTACCAAACTGTGACCTTCGGCCGCCTCTCTCCCAGAGGCAGGTGGAAAGGAGCAGGTGTTTCTGCCCTTCACCGTGCCCCCACACCCTGCGGCCGCGCAGGTCTCCCTCCCAGGCAGGTGCGAAAGTCCCAGGCCACACTTGTGTCTACAAATAGTCATCCACGGGCAGTCAAGAAGGTTCCTTGGTTCTGCCGCTCTCTGAGCAGAAATTGTTGGGGTCGGGGAATAAGAACCAGGAAATCGTTTTTAAGGTTCAAACCCAGTTCTGCTGAGGTCTCAGCTCGAATCTCGGACCACGGGGCCCCGCCTTTCCCGCCACCCTGGCTTGAGGGCAGAGGGGATTTCTGCTGCGGGTTCCGCCTGTGGTCATTGCGTCCCCATTCCGGGCCGTCCGGTCCCAGTCCAATCGGCTCTGGGAGCAGAAGAACACGTGAAAGCTGAACATGGGTTTTCCCTAAATATTGCCTGAGAGCGGGGCGACCCCCAGGCCTGGGCAGGTTCGCGGACCCCAAAGCACCTTCTCTTTCCCCCTCCTCCTGGCCGCTGGCTTCCGCCCCCTCCTACCCTCCCACCGGGTTGCCTCTGATTCCTCCTGGACTCCGATCTTTTCACGCTCTTGTTGGTTTCACTGACATGTTCTTGTCAATTTCAAACGCTTTGTGATTGTAAAAAAAAAAAAATCGAACCGATACGGTCCTACCACTCGCCCTAGTTTCGGAGCCCGGAGCTGTCCTGCGTGTGCGTCCATGTGGAGTGTCCGGGGCTGCGGGCTCGGGCGCACGGTGCCAGCCGAGGGCTGCCCTCCGCTTTTGTGTTAACCGGCGGGCTTCTCGCGGTCCCCGCCGCAGAGGTCACACCCGGCGGGTAACGGCGTGGATACACCGAAGGGTGACTTTGGACACCTTCCCCACACCACAGACTAACGCTTCTGCCCCTACTCCGCCCCTGCTAGAGAAGTAGGAGGCCAGTGGGGGAGGGGGTATTTTCCTGAAGCTCCAGAAAATGACCACGCATTTTAGAGAAAGGTCGTGCCCGCTTCCCAGCCTCACCTAGTCTGGGCTGGGGCCGGGACCCGCCTCCCCACCTTCCCCGCCCCCCCCGCTCTTCAACCTAGCGGAGGGACAGATGCCAGCGCGGTGGAGTCATGCCGCTGGCTTGGGCACCATTGGTCATGCCTGGAACACGCAGCAGCGAGTACGCACATCTGGCGGCTATCCCGGGCGGCTCCGGTCCTGATATGGAGAGAGAGGGCGGGCTGGTGTGTGTCTCAGTGAGCGAGGCTGGGGGAACGCGCCTGGGCTGGCTCCTCCCCGAACTTGCATCACCAGTGCCCCCTCTCTCCACCCGCCTTCGGCCCCGCCTTGGCCCCTCCTCCACCCCCCTTCCTCCGCTCCGTTCGGCCGGTTCTCCCGGGAAGCTATTAATAGCATTACGTCAGCCTGGGACTGGCAACACGGAGTAAACGACCGCGCCGCCAGCCTGAGGGCTATAAAAGGGGTGATGCAACGCTCTCCAAGCCCATGTGTTGTGCTGGTTTCTGTTCATTTAAATCTGTCGGTTGCTGAGACCTAGCGATTCCCTGCCTTTCCCTCCCCATTATGGGGGGTGCCTAGCTTTAA

The R language code used for single-cell data analysis and the ferroptosis pathway geneset were available in GitHub repositories (https://github.com/Xiao851213/SCFA_Ferroptosis_new/blob/main/20240329).

### Cell culture

The human AC16 cardiomyocytes (cat. #C1360, WHELAB, China) and murine HL1 cardiomyocytes (cat. #C2173, WHELAB, China) were cultured in a humidified incubator (5% CO2, 37°C) with the Dulbecco’s modified Eagle’s (DMEM)/F12 1:1 medium (cat. #CB003, Shanghai Epizyme Biomedical Technology Co., Ltd, Shanghai, China) supplemented with 12.5% fetal bovine serum (FBS, cat. #S711-001S, the Lonsera) and penicillin/streptomycin (100 U/mL, 100 U/mL, cat. #CB010, Shanghai Epizyme Biomedical Technology Co., Ltd., Shanghai, China). Cells at 70%–80% confluence were used for subsequent experiments.

### Hypoxia-reoxygenation injury model

The hypoxia-reoxygenation (H/R) model was induced using the AnaeroPack™ (anaerobic cultivation set) with an airtight container (a 2.5 L rectangle jar, Mitsubishi gas chemical, Japanese) (Wen et al., 2021). In detail, cells cultured for 1 day were washed twice with phosphate‐buffered saline (PBS), cultured in sugar and serum‐free DMEM, and then placed into a sealed airtight container that contains an AnaeroPack, the oxygen concentration decreased to <0.1% within 1 hour, and the carbon dioxide concentration was maintained at about 5%. Hypoxia was continued for 10 h and terminated by removing the culture bottle from the airtight container and replacing it with a standard culture medium in a CO_2_ incubator at 37°C for 6 h.

### SCFA exposure

To assess the impact of SCFAs on the viability of cardiomyocytes *in vitro*, AC16 cells were seeded in 96‐well plates for 24 h with DMEM/F12 containing 12.5% FBS. The cells were then treated with either sodium acetate (NaAc, cat. #S116319, Aladdin, Shang, China), sodium butyrate (NaBu, cat. #S102954, Aladdin, Shanghai, China), sodium propionate (NaPr, cat. #S100121, Aladdin, Shanghai, China), or a SCFA mixture (NaAc: NaPr: NaBu ≈ 30:2:1) for 24 h.

The detectable physiological levels of SCFA are in the range of (acetate 0–410 μM; propionate 0–18.3 µM; butyrate 0–81 μM, including blood, cerebrospinal fluid (CSF), breast milk, and urine; Human Metabolome Database, http://www.hmdb.ca/) and the relative levels of the three SCFAs correspond to approximately 30:2:1 for acetate: propionate: butyrate (Yang et al., 2020). The physical and upper physiological levels of concentrations were adopted to obtain a dose-dependent curve. The concentrations of the SCFAs are presented in Table 2.

**TABLE 2:** Short-chain fatty acid concentrations used in this study.

SCFA concentration	C0	C1	C2	C3	C4	C5	C6	C7
Mixture	0	NaAc (3 μM) + NaPr (0.2 μM) + NaBu (0.1 μM)	NaAc (30 μM) + NaPr (2 μM) + NaBu (1 μM)	NaAc (300 μM) + NaPr (20 μM) + NaBu (10 μM)	NaAc (3 mM) + NaPr (0.2 mM) + NaBu (0.1 mM)	NaAc (30 mM) + NaPr (2 mM) + NaBu (1 mM)	NaAc (300 mM) + NaPr (2 mM) + NaBu (1 mM)	NaAc (3 M) + NaPr (0.2 M) + NaBu (0.1 M)
NaAc	0	3 μM	30 μM	300 μM	3 mM	30 mM	300 mM	3 M
NaPr	0	0.2 μM	2 μM	20 μM	200 μM	2 mM	20 mM	200 mM
NaBu	0	0.1 μM	1 μM	10 μM	100 μM	1 mM	10 mM	100 mM

The mixture consists of sodium acetate (NaAc), sodium propionate (NaPr), and sodium butyrate (NaBu) with concentrations in the same column of the table; for example, the C1 mixture = NaAc (3 μM): NaPr (0.2 μM): NaBu (0.1 μM).

### Ferroptosis model induction and assessment

The ferroptosis model was induced by erastin (cat. #S7242, Selleck), a typical ferroptosis inducer (Yan et al., 2022). Erastin was diluted to a 10 mM working stock solution with dimethylsulfoxide (DMSO). AC16 cells were seeded in 96‐well plates for 24 h with DMEM/F12 containing 12.5% FBS, followed by exposure to 10 µM erastin for 24 h (Wu et al., 2023).

A Liperfluo probe (cat. #L248, Dojindo Molecular Technologies, Inc.) was used to evaluate cellular lipid peroxidation. Cells after the indicated treatments were washed with serum‐free DMEM and incubated with 5 μM Liperfluo for 30 min at 37°C (Nakamura et al., 2023). The intracellular Fe^2+^ was detected by the FerroOrange probe (cat. #F374, Dojindo Molecular Technologies, Inc.). Cells were incubated with 1 μM FerroOrange for 30 min at 37°C (Tian et al., 2021). Stained cells were observed using confocal scanning microscopy. The fluorescence of each group was evaluated using ImageJ software.

### CCK-8 assay

Cell viability was assessed using the cell counting kit-8 (CCK-8, cat. #CX001S, Shanghai Epizyme Biomedical Technology Co., Ltd, Shanghai, China). Briefly, cells were incubated with fresh medium (containing 10% CCK-8 reagent) for 2 h. The optical density at 450 nm (OD450) was determined by a microplate reader (BioTek, United States) and normalized to blank wells (cell-free medium with CCK-8 reagent).

### Quantification of mRNA levels

Total RNA was acquired using a TRIzol reagent (cat. #R0016, Beyotime, Shanghai, China) and an RNA extraction kit (cat. #A2010A0402, BioTNT, Shanghai, China). The concentration was analyzed with a Nanodrop 8000 spectrophotometer (Thermo Fischer Scientific), with concentration at 50–120 ng/μL and A260/A280 of 1.8–2.1 for all samples. RNA was converted into cDNA using a reverse transcription kit (Wuhan servicebio Technology CO., LTD, Wuhan, China). Then, RT‐PCR was performed using SYBR Green qPCR Master Mix (Wuhan servicebio Technology CO., Ltd., Wuhan, China); expression was detected using a fast real‐time PCR system (CFX Connect, Bio-rad, CA, United States). Cycle counts for mRNA quantification were normalized to GAPDH. Relative expression (ΔCt) and quantification (RQ = 2^–ΔΔC^) for each mRNA were calculated using the ΔΔCt method. All reactions were performed according to the manufacturer’s instructions. All primers were verified for producing a single specific PCR product via melting curve analysis. The primers used in the study are presented in Table 3.

**TABLE 3:** Primers for RT-PCR

Genes	Primers
*G*APDH	5′-CCTCGTCCCGTAGACAAAATG-3′, 5′-TGAGGTCAATGAAGGGGTCGT-3′
*ATF3*	5′-CGCTGGAGTCAGTTACCGTCAA-3′, 5′-TTCCGGTGTCCGTCCATTC-3′
*VDR*	5′-CTGCCTGACCCTGGTGACTT-3′, 5′- CTTGGTGATGCGGCAATCT-3′
*EGR3*	5′-ACTACAACCTGTACCACCATCCCA-3′, 5′-TGATGGTCTCCAGTGGGGTAAT-3′
*PAX5*	5′-CATCAAGCCAGAACAGACCACA-3′, 5′-TGACAATAGGGTAGGACTGTGGG-3′
*SP1*	5′-AAGATGTTGGTGGCAATAATGGG-3′, 5′-GTTGTTGCTGTTCTCATTGGGTG-3′

### Western blot

Total cellular proteins were extracted using RIPA lysis buffer (cat. #FD008, HANGZHOU FUDE BIOLOGICAL TECHNOLOGY CO. LTD., China), ultrasonic lysis machine (cat. #VCX130, Sonics & Materials, INC. United States), and metal bath (cat. #HB120-S, DragonLab DWB, China). Proteins were separated via electrophoresis on a 4%–20% SDS gel (cat. #36250ES10, YEASEN, China) and transferred to PVDF membranes (cat. #IPVH00010, Millipore, Germany). After blocking with 5% bovine serum albumin (BSA, cat. #V908933, Merk, Germany) for 1 h, The PVDF membranes were incubated with primary antibodies, including anti-GAPDH (1:5000, cat. #A19056, ABclonal, China) (Bian et al., 2024), anti-ATF3 (1:1000, cat. #A13469, Abclonal, China) (Li et al., 2023; Liu et al., 2023), and anti-GPX4 (1:1000, cat. #CL488-67763, PTG, China) (Wang L. et al., 2022) antibodies at 4°C for >10 h. Subsequently, the membranes were incubated with the HRP-conjugated Goat anti-Rabbit/Mouse IgG (H + L) (cat. #AS014 & AS003, Abclonal, China) for 1 hour at room temperature. The protein bands were visualized with a Fdbio-Dural ECL Chemiluminescence Kit (cat. #FD8020, HANGZHOU FUDE BIOLOGICAL TECHNOLOGY CO. LTD., China) and imaged.

## Results

### The relationship between ATF3 and ferroptosis pathway in ischemic heart

**FIGURE 1:**
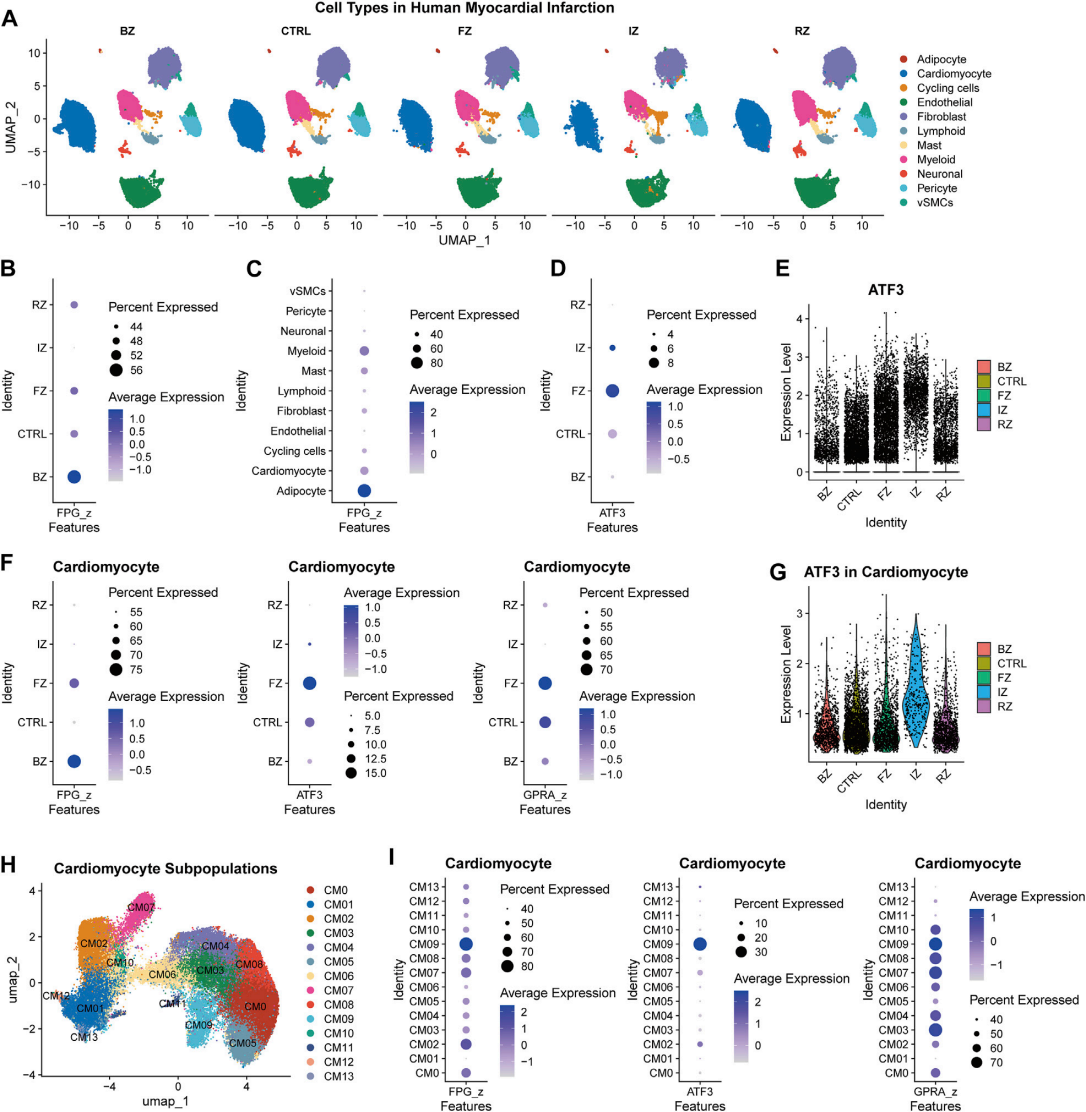
The relationship between ATF3 and ferroptosis pathway in ischemic heart **(A)**, Uniform manifold approximation and projection (UMAP) embedding of the single-nuclear RNA-seq data of myocardial infarction heart tissue derived from the spatial multi-omic atlas (sma) study, titled “Spatial multi-omic map of human myocardial infarction” (Christoph Kuppe et al.). Cell types were illustrated in different cardiac regions (BZ, border zone; CTRL, control samples; FZ, fibrotic zone; IZ, ischaemic zone; RZ, remote zone) **(B,C)**, Dot plot illustrating the expression of ferroptosis pathway geneset (*FPG*) among myocardial infarction regions **(B)** and cell types **(C) (D)**, The expression of ATF3 among myocardial infarction regions **(E)**, Violin plot of ATF3 expression level across myocardial infarction regions **(F)**, The expression of ferroptosis pathway geneset (*FPG*), ATF3, and geneset of potential regulators for ATF3 (*GPRA*) in different regions of cardiomyocytes **(G)**, Violin plot of ATF3 expression in different regions of cardiomyocytes **(H)**, UMAP of 64,510 cardiomyocytes derived from the sma study **(I)**, The FPG, ATF3, and GPRA expression across cardiomyocyte subclusters.

Our research delves into the crucial topic of the ferroptosis pathway genes and the pivotal role of ferroptosis regulator ATF3 in myocardial infarction. To create a comprehensive ferroptosis pathway geneset (FPG), we combined the genes in KEGG hsa04216 and wikipathways WP431. We then calculated the z score of this ferroptosis pathway geneset across cardiac regions and cell types in the published spatial multi-omic atlas dataset (the sma dataset) (Kuppe et al., 2022). This dataset provides an integrative high-resolution map of human cardiac remodeling after myocardial infarction using single-nucleus RNA sequencing (snRNA-seq), single-nucleus chromatin accessibility, and spatial transcriptomic profiling method. The dataset includes 31 samples from 23 individuals, including four non-transplanted donor hearts as controls (CTRL), and samples from tissues with necrotic areas (ischaemic zone (IZ) and border zone (BZ)) and the unaffected left ventricular myocardium (remote zone (RZ)) of patients with acute myocardial infarction. Nine human heart specimens at later stages after myocardial infarction that exhibited ischaemic heart disease were defined as fibrotic zone (FZ) samples. The snRNA-seq part of the sma dataset (191,795 cells included) was extracted for analysis in this study. Figure 1A illustrates the whole cells, identified cell types, and region sources of the sma snRNA-seq dataset. Potential regulators of ATF3 were predicted via the PROMO database (Supplementary Figure S1A) and combined as a geneset of potential regulators for ATF3 (*GPRA*).

In the sma snRNA-seq dataset, BZ samples have the largest proportion of cells with upregulated *FPG* (Figure 1B). When divided by cell types, *FPG* was enriched in adipocytes, myeloid, cardiomyocytes, and mast cells (Figure 1C). The FZ tissue has the most significant proportion of ATF3^+^ cells (Figure 1D). Elevated expression of ATF3 was also observed in IZ cells (Figure 1E). The relative enrichment of *FPG*, ATF3, and *GPRA* for different cell types differed among region groups (Supplementary Figure S2). In the BZ, there was the same elevation trend of *FPG*, ATF3, and *GPRA* in cardiomyocytes, adipocytes, and cycling cells [a cluster with enriched cell-cycle marker gene MKI67 and showed a high score of cell-cycle G2/M and S phases (Kuppe et al., 2022)]. This indicates the involvement of ATF3 and its potential transcription factors in the ferroptosis of cardiomyocytes, adipocytes, and cycling cells during post-MI cardiac remodeling.

In cardiomyocytes, *FPG* was upregulated in injury-related cells (BZ and FZ), while ATF3 and GPRA were mostly enriched in FZ (Figure 1F). The elevation of ATF3 was also found in IZ cardiomyocytes (Figure 1G). To identify the cardiomyocyte subpopulation that is critical to ferroptosis, we extracted cardiomyocyte data from the sma snRNA-seq dataset, corrected the batch effect (Supplementary Figure S3A, B), and re-clustered according to cell density on the Uniform manifold approximation and projection (UMAP) plot to recognize the subclusters of each region (Supplementary Figure S3C). Cardiomyocytes were clustered into 14 subpopulations, which are CM0-CM13 (Figure 1H; Supplementary Figure S4). The *FPG* was enriched in the FZ cluster CM09 (top marker genes: *ABRA*, *DDIT3*, and *OTUD1*) and the BZ cluster CM02 (top marker genes: UBASH3B, C4orf54, NRXN3). The co-enrichment of FPG, ATF3, and GPRA were also observed in cardiomyocytes’ CM09 and CM02 clusters (Figure 1I; Supplementary Figure S4B). In conclusion, ATF3 may involved but partially regulates ferroptosis pathway genes in myocardial infarction.

### SCFA regulates ferroptosis in the physiological and pathophysiological condition

We then adopted human (AC16 cell line) and murine (HL1 cell line) cardiomyocytes to study the effect of SCFAs on ferroptosis and ATF3 expression at physiological conditions, 1 h-hypoxia exposure, hypoxia-reoxygenation (H/R) model, and erastin-induced ferroptosis model. A dose-dependent toxic experiment was run to detect the suitable dose for SCFA treatment. The concentration of acetate, propionate, butyrate, and SCFA mixture was divided into seven levels (Table 2). The 24-h treatment of SCFAs with the C6 and C7 concentrations decreased cell viability (Supplementary Figure S5). In the 1-h hypoxia model, SCFAs (sodium propionate (NaPr), sodium butyrate (NaBu), and mixture) significantly and consistently decrease cardiomyocyte viability (Supplementary Figure S5B). The C5 level concentration (the maximum dose that does not reduce cell viability) was adopted for the following experiments.

**FIGURE 2:**
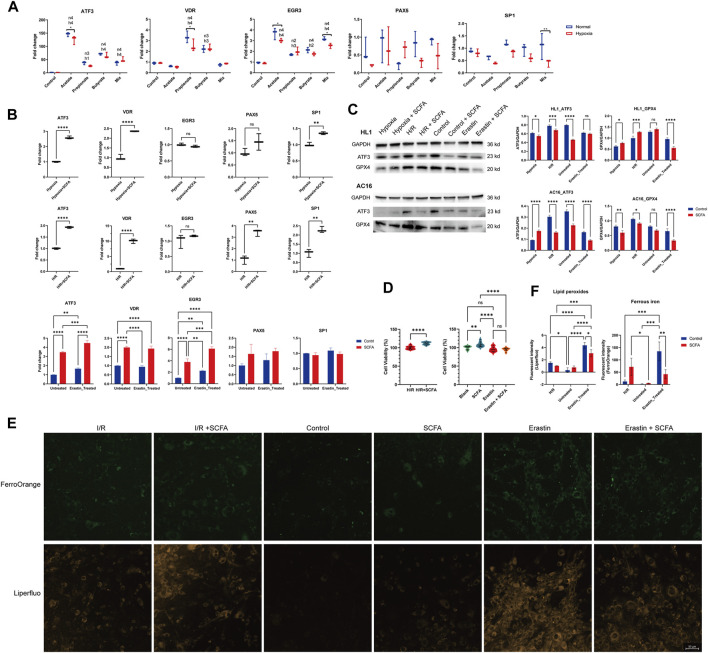
Short-chain fatty acids (SCFAs) regulate ferroptosis and ferroptosis-related genes **(A)**, Murine HL1 cardiomyocytes were treated for 24 h with SCFAs and either 1-h hypoxia stimulation or normoxic conditions. Acetate: 30 mM, propionate: 2 mM, butyrate: 1 mM, SCFA mixture: 30 mM acetate, 2 mM propionate, and 1 mM butyrate. *p < 0.05 and **p < 0.01 for comparisons between the hypoxia and normal conditions groups. h1: *p* < 0.05, h2: *p* < 0.01, h3: *p* < 0.001, h4: *p* < 0.0001, hypoxia group gene expression fold change compared to control. n1: *p* < 0.05, n2: *p* < 0.01, n3: *p* < 0.001, n4: *p* < 0.0001, normal condition group gene expression fold change compared to control **(B)**, Human AC16 cardiomyocytes were treated for 24 h with SCFA (a mixture containing 30 mM acetate, 2 mM propionate, and 1 mM butyrate) and either 1-h hypoxia stimulation (upper panel), H/R (10-h hypoxia plus 6-h re-oxygen, middle panel), or erastin-induced ferroptosis model (10 µM erastin for 24 h, lower panel) **(C)**, HL1 (upper panel) and AC16 cells (lower panel) treated for 24 h with SCFA (a mixture containing 30 mM acetate, 2 mM propionate, and 1 mM butyrate) followed by 1-h hypoxia stimulation, H/R (10-hour hypoxia plus 6-hour re-oxygen), or erastin-induced ferroptosis model (10 µM erastin for 24 h). The ATF3 and GPX4 protein levels were detected via western blot. The density of the plot was quantitated via the ImageJ software. **(D)**, The effect of SCFA on cell viability was tested using the CCK8 method in H/R (10-h hypoxia plus 6-h re-oxygen) and the erastin-induced ferroptosis (10 µM erastin for 24 h) model **(E)**, Effect of SCFA on ferroptosis index in H/R (10-hour hypoxia plus 6-hour re-oxygen) and the erastin-induced (10 µM erastin for 24 h) ferroptosis. Representative Liperfluo and FerroOrange staining images are presented (scale bar: 50 μm) **(F)**, Semiquantitative analysis of the fluorescence intensity of lipid peroxides (detected by Liperfluo) and ferrous iron (detected by FerroOrange). For **(A–D,F)**, Data are expressed as the mean ± SD. Ns, no significance, *p < 0.05, **p < 0.01, ***p < 0.001, ****p < 0.0001, calculated by either t-test or two-way ANOVA with Tukey’s post hoc test via the GraphPad software. *n* = 3 (3 independent experiments).

SCFA treatment can promote the mRNA expression level of *ATF3*, either in mice (normal and 1h-hypoxia model, Figure 2A) or human cardiomyocyte cell line (normal, 1h-hypoxia exposure), H/R exposure (10-h hypoxia plus 6-h re-oxygen), and erastin-induced ferroptosis model, Figure 2B). The potential promoters of ATF3 (*VDR, EGR3*, *PAX5*, and *SP1*) were also affected by 24-h SCFA exposure. In the murine cardiomyocyte cell line, NaPr and NaBu upregulated *VDR* under both normal and hypoxic conditions, while hypoxia attenuated the *VDR* upregulation by NaPr. *EGR3* was upregulated by either a single or a mixture of SCFA. The effect of sodium acetate (NaAc) and the SCFA mixture on *EGR3* was attenuated by 1-h hypoxia exposure (Figure 2A). In the human cardiomyocyte cell line, VDR and SP1 were upregulated by SCFA mixture in 1-h hypoxia exposure and H/R model (Figure 2B, upper & middle panel). SCFA mixture promotes the expression of PAX5 in the H/R model but not the 1-h hypoxia model (Figure 2B, upper & middle panel). In the erastin-induced ferroptosis model, *VDR* and *EGR3* were increased under SCFA mixture stimulation (Figure 2B, lower panel).

We also detected the effect of SCFA on the ATF3 protein level. The results were totally opposite to those of the mRNA level. SCFA decreased the ATF3 protein in nearly all cases except the 1 h-hypoxia model of the human cardiomyocyte AC16 cell line (Figure 2C). The protein expression of anti-ferroptosis markers, glutathione peroxidase 4 (GPX4), was inhibited by the SCFA mixture except in the 1h-hypoxia model of the mouse cardiomyocyte HL1 cell line.

SCFA treatment can increase the cell viability of AC16 cardiomyocytes in H/R exposure (*p* < 0.0001) but not the erastin-induced ferroptosis model (*p* = 0.9984) (Figure 2D). To confirm the occurrence of ferroptosis, we performed Liperfluo staining and FerroOrange staining in the H/R model and erastin-induced ferroptosis model with or without SCFA rescue (Figures 2E, F). Liperfluo staining showed obvious lipid peroxidation in response to H/R or erastin stimulation. This effect in the erastin stimulation model was rescued by SCFA treatment. In the H/R model, a decrease in lipid peroxides was observed in the SCFA-managed group but without statistical significance. The fluorescence intensity of FerroOrange, a Fe^2+^-specific probe, increased sharply upon erastin stimulation. SCFA treatment can reverse erastin-induced Fe^2+^ increase but may strengthen the Fe^2+^ in the H/R model.

## Discussion

In this study, we found the upregulation of the ferroptosis pathway geneset (derived from the KEGG hsa04216 and wikipathways WP4313) and ATF3 in infarction-stimulated cardiac cells (border zone, ischemic zone, and fibrotic zone), particularly the cardiomyocytes. The ATF3 and some of its potential transcription factors (*VDR, EGR3*, *PAX5*, and *SP1*) can be regulated by SCFA. SCFA can attenuate ischemia-reperfusion cell death and erastin-induced lipid peroxidation cardiomyocytes. SCFA treatment can also reverse erastin-induced Fe^2+^ increase but may strengthen the Fe^2+^ in the H/R model. We also precisely defined a ferroptosis subcluster of cardiomyocytes (ABRA^+^DDIT3^+^OTUD1^+^ CM09).

The ATF3, a member of the activator protein 1 (AP-1) transcription factor family, plays a crucial role in various cellular processes, including cell differentiation, apoptosis, proliferation, inflammation, and responses to cellular stress (Hai and Hartman, 2001). It has been noted that ATF3 promotes ferroptosis9, 10 and improves pathological cardiac fibrosis (Wang B. et al., 2022). However, it has also been implicated that ATF3 expression in cardiomyocytes preserves homeostasis in the heart and controls peripheral glucose tolerance (Kalfon et al., 2017). Otherwise, elevated ATF3 can inhibit cardiomyocyte ferroptosis triggered by erastin and RSL3 (Liu H. et al., 2022). In our study, SCFA may inhibit cardiomyocyte ferroptosis via the regulation of ATF3 expression in either H/R injury or erastin-induced ferroptosis.

The upregulation of these genes in response to SCFAs suggests that SCFAs can potentially influence various downstream cellular processes such as ferroptosis. Ferroptosis is closely linked to specific molecular pathways associated with lipid peroxidation, which can be triggered by intracellular iron supplementation and inhibition of the synthesis of GSH (Kang et al., 2023). Previous research has indicated that NaAc can reverse the nicotine-induced elevation of plasma GSH levels, (Dangana et al., 2020), while NaBu has been shown to exacerbate lipid peroxidation (Oyabambi and Olaniyi, 2023). Consequently, SCFAs have the potential to either promote cell ferroptosis via the GSH inhibition effect or attenuate ferroptosis via the anti-inflammatory and anti-oxidative stress effect. Butyrate could ameliorate ferroptosis in ulcerative colitis by modulating the Nrf2/GPX4 signal pathway and improving the intestinal barrier (Chen et al., 2024).

In our study, SCFA has nearly the opposite effect on the mRNA and protein levels of ATF3 and GPX4. This indicates the post-translational regulation function of SCFA, which is consistent with a previously published article that butyrate could reduce the expression of inflammatory genes via the inhibition of mRNA-stabilizing proteins (Torun et al., 2019). SCFA presented with the attenuation of H/R-induced cell death and erastin-induced cardiomyocyte ferroptosis, proved by the change of cell viability, ferrous iron, and lipid peroxides. While no effect is observed in Supplementary Figure S5A; Figure 2D (right graph) shows that SCFAs increase viability. However, the differences in cell viability between control and SCFA-treated groups are minimal, indicating the limited effect in our studied models and heterogeneity among different experiments.

Our previous study demonstrated that SCFAs exert a negative cardiac inotropic effect both *in vitro* and *in vivo*, providing evidence of their direct impact on cardiac tissue (Poll et al., 2021). NaBu has been reported to offer protection against cardiac I/R injury and induce changes in gene expression within the cardiac tissue. Specifically, these gene expression alterations were observed in pathways related to “signaling molecules and interaction,” “immune system,” “cell growth and death,” and “global and overview maps,” including pathways associated with antigen processing and presentation (Yu et al., 2021). Another study published in 2016 demonstrated that NaBu can protect against oxidative stress in HepG2 cells (Xing et al., 2016). These findings strengthen the stability of our study.

The unique elevated ferroptosis level in adipocytes of cardiac tissue was observed in this study, which was not reported before. However, it has been reported that high-altitude hypoxia exposure can induce iron overload and ferroptosis in adipose tissue (Zhang et al., 2022). Since the adipose tissue is a crucial regulator secreting various bioactive factors signaling to myocardial cells, (Liu X. et al., 2022), ferroptosis pathway dysregulation in cardiac adipocytes may play critical roles in responding to cardiac ischemic and I/R injury.

There were some limitations in this study. First, whether SCFA attenuated cardiomyocyte H/R injury via inhibition of ferroptosis still needs to be explored. Second, whether the effects of SCFA on I/R injury and ferroptosis rely on ATF3 regulation remains unknown. On the other hand, there are two direct receptors of SCFA, G-protein coupled receptor 41 (GPR41) and GPR43. The role of GPR41/43 in SCFA benefits has not been studied. These issues will be investigated in future research.

## Conclusion

In the heart of myocardial infarction, the ferroptosis pathway is elevated in cardiomyocytes and adipocytes injury-related cardiac regions (border zone, ischemic zone, and fibrotic zone), as well as the ATF3. SCFA can regulate lipid peroxidation and ferrous iron induced by either hypoxia-reoxygenation or erastin. SCFA can promote the stress-responsive and ferroptosis gene ATF3 at the mRNA level but inhibit the protein level. We also identified a distinct subcluster of cardiomyocytes exhibiting a high ferroptosis pathway expression level. These findings shed light on potential targets of SCFAs involved in ferroptosis and their role in conferring protection against cardiac ischemic injury.

## Data Availability

Publicly available datasets were analyzed in this study. This data can be found here: https://cellxgene.cziscience.com/collections/8191c283-0816-424b-9b61-c3e1d6258a77.

## Supplementary material

SUPPLEMENTARY FIGURE S1 The screening process to identify transcription factors (TFs) associated with ATF3 **(A)**, Identification of the TFs predicted to associate with the promoter of ATF3, as determined by PROMO software. A total of 83 transcription factors were identified **(B)**, The binding site of VDR, EGR3, PAX5, and SP1 on the promoter region of ATF3.

SUPPLEMENTARY FIGURE S2 The heterogeneity of ferroptosis pathway genes, ATF3, and its promoters geneset **(A)**, Cell types heterogeneity of ferroptosis pathway genes (FPG) in different cardiac regions of the sma dataset **(B)**, Cell types heterogeneity of ATF3 in different cardiac regions of the sma dataset **(C)**, The geneset of potential regulators for ATF3 (*GPRA*) in different cardiac regions of the sma dataset.

SUPPLEMENTARY FIGURE S3 The cardiomyocyte in the published sma dataset **(A)**, The sma single-nuclear cardiomyocytes data before batch correction **(B)**, The sma single-nuclear cardiomyocytes data after batch correction **(C)**, Density estimation of the number of cardiomyocyte nuclei split by the cardiac zone.

SUPPLEMENTARY FIGURE S4 The cardiomyocyte subclusters of human myocardial infarction tissue **(A)**, UMAP of cardiomyocyte subclusters in different zones of the human heart. RZ, remote zone; BZ, border zone; IZ, ischemic zone; FZ, fibrotic zone **(B)**, Top marker genes of the cardiomyocyte subclusters **(C,D)**, Distribution of cardiomyocyte subclusters in different groups **(C)** and samples **(D)**.

SUPPLEMENTARY FIGURE S5 Dose-dependent effect of SCFAs on cardiomyocyte viability. Cell viability of AC16 cells treated with different concentrations of SCFAs (24 h) at normal condition **(A)** or with 1-h hypoxia exposure **(B)**. N = 3. Data are expressed as the mean ± SD. Significance was calculated using one-way ANOVA with Tukey’s post hoc test or the t-test. *p*-values < 0.05 were considered statistically significant. **p* < 0.05, ***p* < 0.01, ****p* < 0.001, *****p* < 0.0001, compared to C0 concentration. SCFA concentration (C0–C7) is presented in Table 2.
