# Supplementary figures and images for: Short-chain fatty acids regulate erastin-induced cardiomyocyte ferroptosis and ferroptosis-related genes
Source: Front Pharmacol. 2024 Jul 12;15:1409321. doi: 10.3389/fphar.2024.1409321 (PMC11272585; doi:10.3389/fphar.2024.1409321)

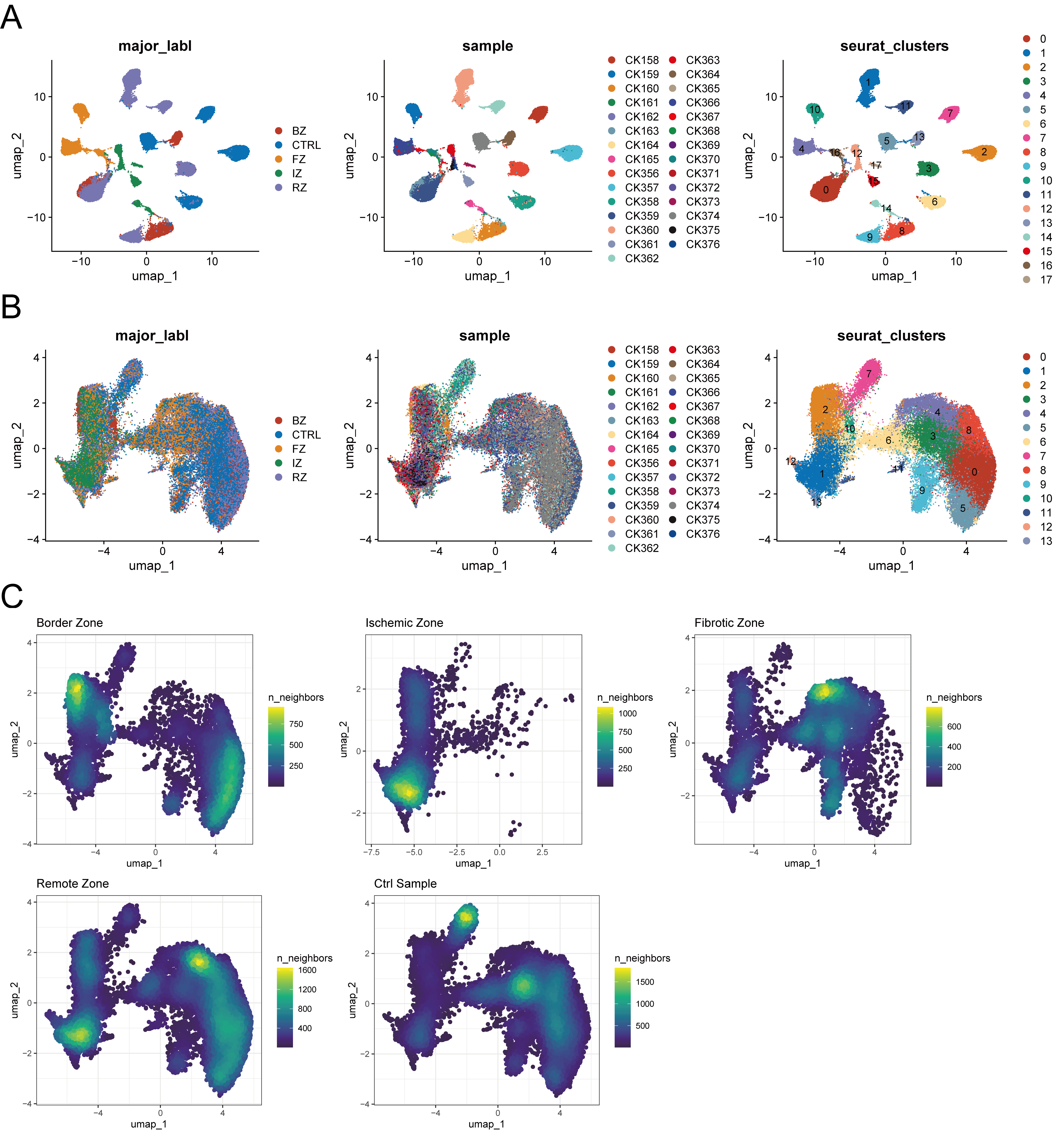

Supplement: Supplementary file 1 [file Image3.TIF]

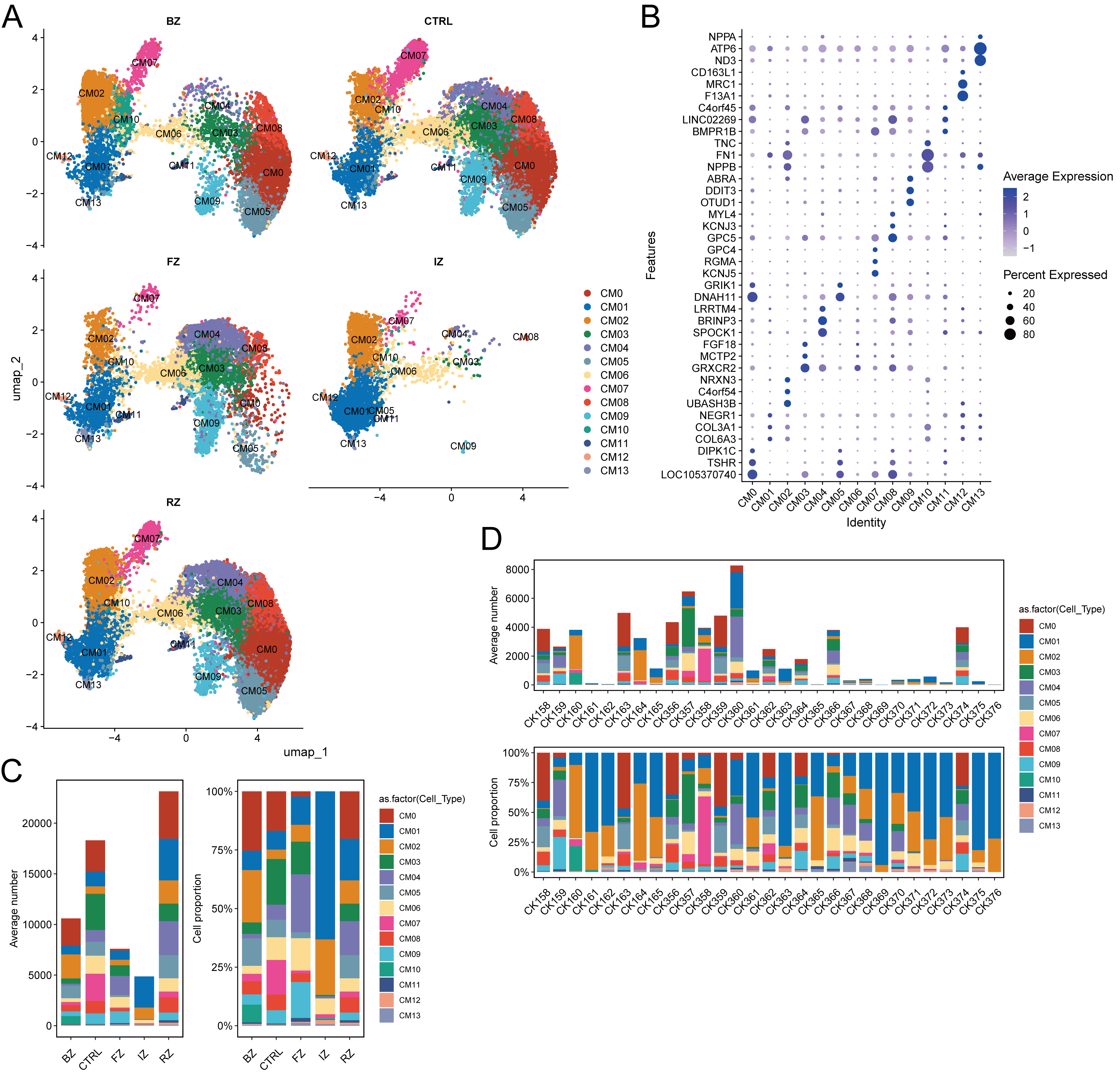

Supplement: Supplementary file 2 [file Image4.TIF]

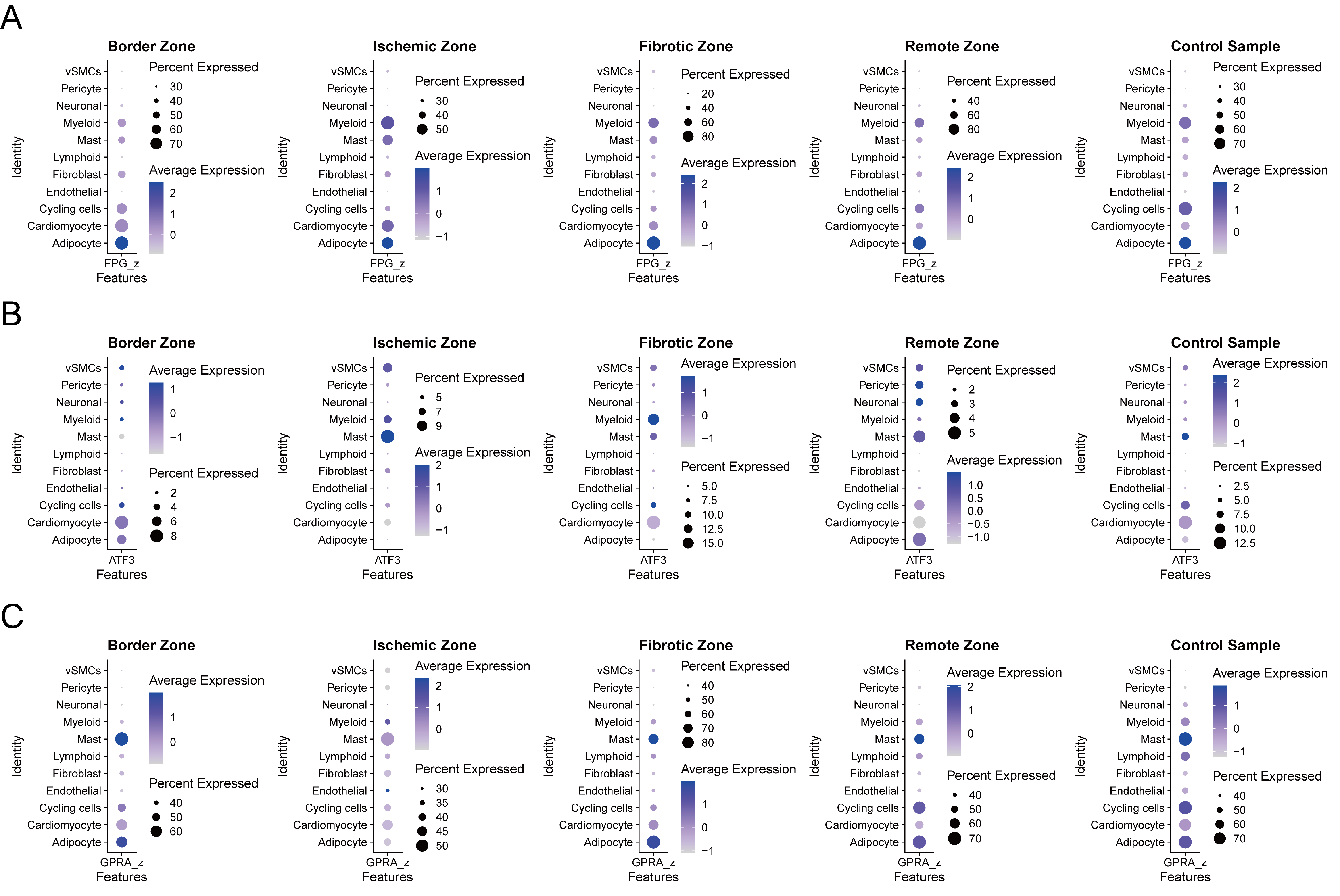

Supplement: Supplementary file 3 [file Image2.tif]

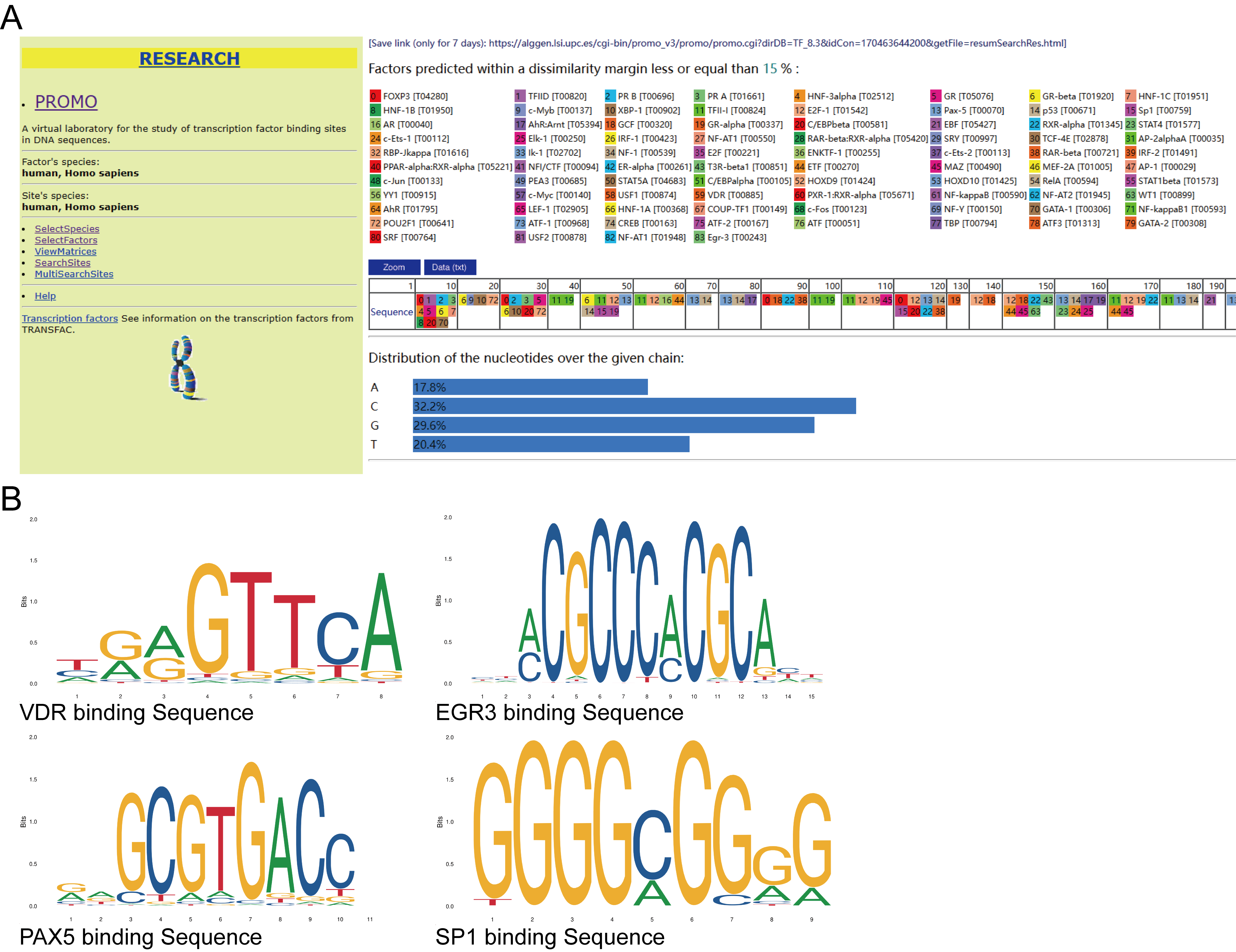

Supplement: Supplementary file 4 [file Image1.TIF]

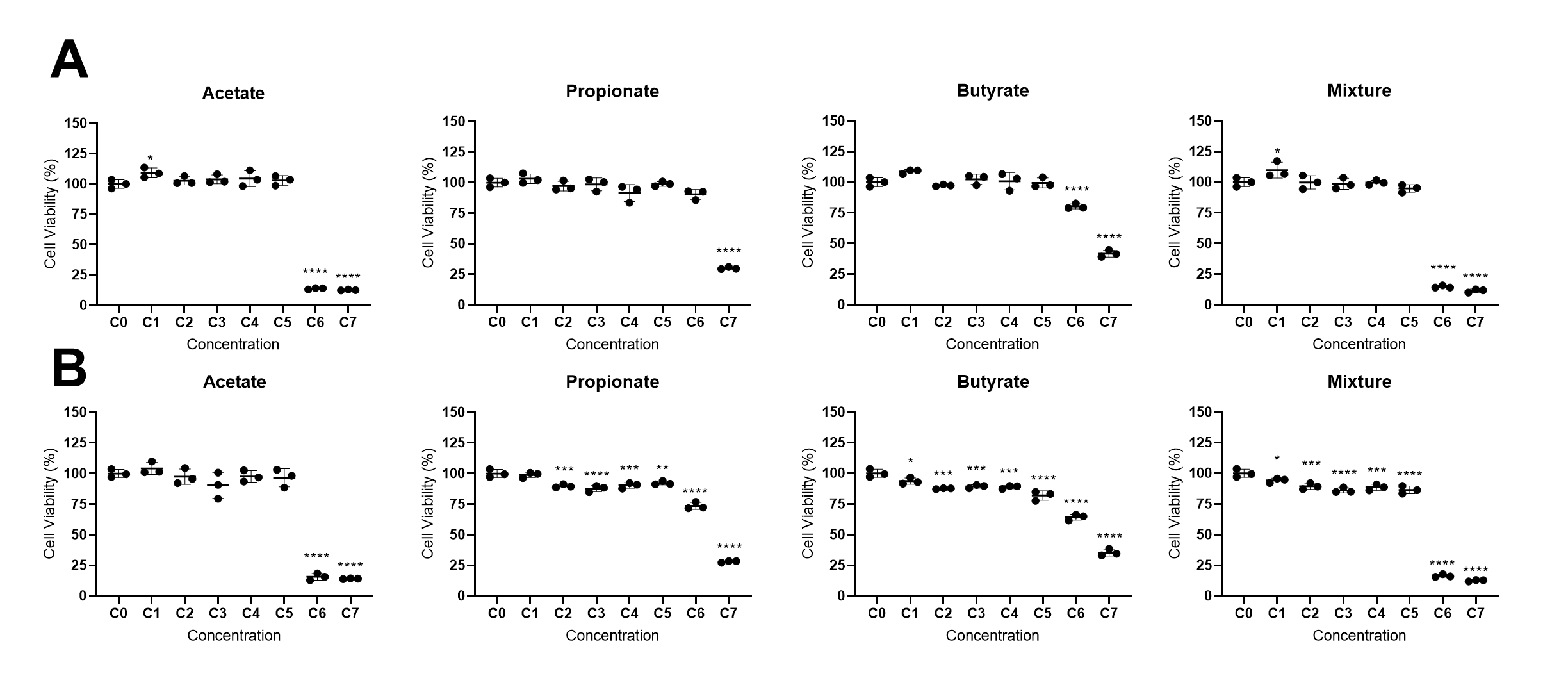

Supplement: Supplementary file 5 [file Image5.TIF]
